# Supplementary material for: Predictive performance of oximetry in detecting sleep apnea in surgical patients with cardiovascular risk factors
Source: PLoS One. 2021 May 6;16(5):e0250777. doi: 10.1371/journal.pone.0250777 (PMC8101727; doi:10.1371/journal.pone.0250777)
Supplement: Attachment — Submitted filename: Response to Reviewers .docx [file pone.0250777.s002.docx]

**To**

**James Andrew Rowley**

**Academic Editor**

**PLOS ONE**

**Re: Predictive Performance of Oximetry in Diagnosis of Sleep Apnea in Surgical Patients with Cardiovascular Risk Factors**

**We are delighted to hear of the possible acceptance of our manuscript for publication in PLOS ONE. Thank you for your interest in our work. We appreciate the constructive comments of the reviewer and the editor. We are grateful for the opportunity to revise the manuscript. Please find below a point-by-point response detailing the revision and the respective changes made in the manuscript. Changes are bold in the manuscript. We hope that the revision of our manuscript would be accepted for publication.**

**We have also provided sample of data for two variables involved in the study. I reported receiving grants from the Ontario Ministry of Health and Long-term Care, University Health Network Foundation. STOP-Bang questionnaire: proprietary to University Health Network.** **This does not alter our adherence to PLOS ONE policies on sharing data and materials.**

**Sincerely yours,**

**Frances Chung MBBS FRCPC**

**Professor, Dept of Anesthesiology and Pain Medicine, University of Toronto,
ResMed Research Chair of Anesthesiology, Sleep, and Perioperative Medicine,**

**Toronto Western Hospital, University Health Network.**

**Format**

3. In your Methods section, please provide additional information about the participant recruitment method and the demographic details of your participants. Please ensure you have provided sufficient details to replicate the analyses such as: a) a description of how participants were recruited, and b) descriptions of where participants were recruited and where the research took place.

**Patients undergoing major elective non-cardiac surgery were approached for the study. Patients were recruited if they had at least one risk factor of postoperative vascular events. Details of the methodology can also be found in the protocol of the original study. We now added more information in the methods.**

**Chan MT, Wang CY, Seet E, Tam S, Lai HY, Walker S, Short TG, Halliwell R, Chung F, POSA Investigators. Postoperative vascular complications in unrecognised Obstructive Sleep apnoea (POSA) study protocol: an observational cohort study in moderate-to-high risk patients undergoing non-cardiac surgery. BMJ open. 2014 Jan 1;4(1).**

**Editor Comment:**

My major comment is that it is unclear what this study is to the literature. The cohort is one of the largest to be studied, was multi-center and included patients with CV risk factors. The authors need to convince me that there is some truly unique about this data set. Therefore, what would tremendously help is outcomes data. Since the dataset is from a large cohort study of surgery, the authors would have post-surgical outcomes, either cardiac or pulmonary. The authors could determine if ODI alone (ie, not in conjunction with the AHI) is predictive of post-surgical complications. Such an analysis would greatly strengthen the data and provide more than just another validation of oximetry v. HSAT.

**Our study is different from the previous studies of ODI that correlated with a type 3 device. The study includes a large representative sample of ethnically diverse (Chinese, Indians, Caucasian, Malaysian) patients from eight clinical sites in five countries undergoing non-cardiac surgery. Additionally, our study involved relatively older patients (inclusion criteria ≥45 years) with mean age of 67 years. Previous studies mostly involved younger patients (inclusion criteria ≥18 years) who were referred to sleep clinics. Thus, the patients that were referred to sleep clinics were pre-screened. Our study recruited surgical patients that had one cardiovascular risk factor for both sleep study and oximetry. These patients were not pre-screened for symptoms of sleep apnea. Thus, it is different from previous studies that correlated ODI with AHI in patients that were pre-selected and were referred to sleep clinics.**

**Our study is a post hoc analysis of previous work. The original study by Chan et al (2019) already showed that OSA was associated with higher risk of cardiovascular outcomes. It is to be noted that there is a strong correlation between AHI from apnea link and ODI from oximetry. In our previous publication (JAMA 2019), we used AHI to predict post-surgical outcomes. We expect to have similar findings if we use ODI to predict post-surgical outcomes/complications. We agree with the editor that we should examine the post-surgical outcomes using ODI in a future study.**

**Chan MT , Wang CY, Seet E, …Chung F. Association of unrecognized obstructive sleep apnea with postoperative cardiovascular events in patients undergoing major noncardiac surgery. JAMA 2019;321:1788-98.**

Minor comments
1. The authors included patients with CV risk factors but unclear if pulmonary risk factors were an inclusion or exclusion. Key as COPD is a risk factor for CV disease.

**Patients with COPD were included in the study as shown in the Table 1.**

2. Why did the authors exclude patients requiring mechanical ventilation > 2 days? Given my question about outcomes, very key to include ALL patients in such an analysis. Does this group have pre-surgery HSAT/ODI data?’

**Patients on mechanical ventilation for > 2 days are seriously sick. They would require intubation, mechanical ventilation, and higher FiO_2_. The mechanical ventilation > 2 days likely prevents or modifies the severity of OSA and affects the results of ODI from oximetry. Since these patients were excluded, we do not have any data for this group.**

3. Results: the authors present data in two different ways: either to no decimal place or to the 10th decimal place; should be uniform in both tables and would suggest to the 10th decimal place.

**Thank you. We have changed the data to the 10^th^ decimal place.**

4. Table 2: what is respiratory event index?

**Respiratory event index is one of the variables from ApneaLink. We have removed it to avoid the confusion**
5. Page 14: the paragraph on COVID seems to be added just because we’re in the midst of the pandemic. Not sure needed for the greater picture of this study.

**Oximetry is a screening tool that requires minimum instruction. Regardless of the COVID pandemic, oximetry provides a quick and accessible approach in screening patients with risk of sleep apnea. Even after the pandemic, there will be hesitancy in patient facing activities. Oximetry can be used a screening tool in such instances.**

6. While it appears that ODI>15 is good for both moderate-severe and severe AHI cutoff if use ODI>15, will not be able to distinguish between the two. This should be clarified.

**This is correct that ODI** **≥ 15 cannot distinguish between moderate-severe or severe sleep apnea. In the clinical setting if someone has ODI ≥15 , they can be considered as a ‘high risk’ for both moderate-severe or severe sleep apnea. Based on the risk, they can be referred for in-lab polysomnogram for further diagnosis. We have clarified this in the discussion.**

**Reviewer #1:** Thank you for this well-written and excellent manuscript on HSAT vs pulse-oximetry in a preoperative population. This is an important subject considering the increasingly difficult access of preoperative PSG services.

**Thank you for the nice comments for the recognition of the significance of the study.**

My primary concern is mostly a wordsmithing concern. Language throughout the manuscript refers to the 'diagnostic' performance of ODI from pulse oximetry. This is inaccurate, as OSA cannot be diagnosed solely from oxygen desaturation. I would prefer language throughout the manuscript be revised to reflect 'predictive' performance of ODI for OSA.

**Thank you for the comment. We have replaced the word diagnostic with predictive.**

My biggest methodological question is that you report hypopnea was defined as reduction in airflow for ≥30% for ≥10s from baseline. You also state you used AASM criteria for reporting AHI. Does this mean you used a 3% oxygen desaturation for hypopneas? Please specify.

**Yes, we used 3 % oxygen desaturation for hypopnea. Statement has been added in the methods section.**

A few minor methodological questions:
1. "1,218 at-risk surgical patients without prior risk of sleep apnea." Do you mean patients without prior diagnosis here?

**Prior risk is a typo and should be ‘prior diagnosis’. We have changed it in the abstract methods.**

**One of the exclusion criteria was patients with a previous diagnosis of obstructive sleep apnea or any sleep-related breathing disorder.**

2. "All data was processed by a technician blinded to the study." What was the technician blinded to?

**Technician was blinded to the clinical data, and the STOP-Bang score of the study. Statement has been edited in the methods.**
